# Ethyl 2-(2,6-difluoro­benz­yloxy)-4-(4,6-dimethoxy­pyrimidin-2-yl)quinoline-3-carboxyl­ate

**DOI:** 10.1107/S1600536809030499

**Published:** 2009-08-08

**Authors:** Yuan-xiang Li

**Affiliations:** a Key Laboratory of Hunan Province for the Study and Utilization, of Ethnic Medicinal Plant Resources, Huaihua University, Huaihua 418008, People’s Republic of China

## Abstract

In the title compound, C_25_H_21_F_2_N_3_O_5_, the pyrimidine and difluoro­benz­yloxy rings are twisted away from the central quinoline ring system, making dihedral angles of 54.6 (1) and 74.1 (1)°, respectively. A weak C—H⋯O inter­action links symmetry-related mol­ecules, forming a pseudo-dimer. π–π inter­actions between the quinoline rings of symmetry-related mol­ecules [centroid–centroid distance = 3.5479 (10) Å] link these dimers into chains parallel to [101]. Weak C—H⋯π inter­actions join adjacent chains, forming a two-dimensional layer parallel to (101).

## Related literature

Pyrimidinylbenzoates are highly effective herbicides with acetohydr­oxy acid synthase (AHAS) as target, see: Duggleby & Pang (2000 ▶). For related structures, see: Li & Huang (2007 ▶); Li & Wang (2007 ▶); Li *et al.* (2006 ▶).
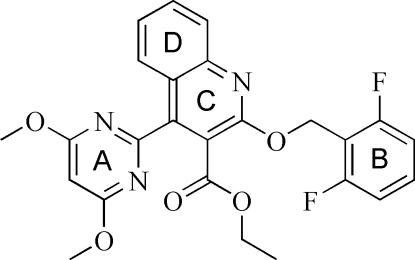


## Experimental

### 

#### Crystal data

C_25_H_21_F_2_N_3_O_5_
*M* *_r_* = 481.45
Triclinic, 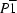

*a* = 9.6623 (6) Å
*b* = 10.7044 (6) Å
*c* = 11.3856 (7) Å
α = 84.466 (1)°
β = 82.251 (2)°
γ = 81.394 (1)°
*V* = 1150.21 (12) Å^3^
*Z* = 2
Mo *K*α radiation
μ = 0.11 mm^−1^
*T* = 297 K
0.20 × 0.20 × 0.10 mm

#### Data collection

Bruker SMART APEX CCD area-detector diffractometer
Absorption correction: multi-scan (*SADABS*; Sheldrick, 1997 ▶) *T*_min_ = 0.959, *T*_max_ = 0.989
12431 measured reflections
4954 independent reflections
3541 reflections with *I* > 2σ(*I*)
*R*_int_ = 0.035

#### Refinement

*R*[*F* ^2^ > 2σ(*F* ^2^)] = 0.052
*wR*(*F* ^2^) = 0.170
*S* = 1.06
4954 reflections
319 parameters
H-atom parameters constrained
Δρ_max_ = 0.30 e Å^−3^
Δρ_min_ = −0.23 e Å^−3^

### 

Data collection: *SMART* (Bruker, 2001 ▶); cell refinement: *SAINT* (Bruker, 2001 ▶); data reduction: *SAINT*; program(s) used to solve structure: *SHELXS97* (Sheldrick, 2008 ▶); program(s) used to refine structure: *SHELXL97* (Sheldrick, 2008 ▶); molecular graphics: *PLATON* (Spek, 2009 ▶); software used to prepare material for publication: *PLATON*.

## Supplementary Material

Crystal structure: contains datablocks global, I. DOI: 10.1107/S1600536809030499/dn2477sup1.cif

Structure factors: contains datablocks I. DOI: 10.1107/S1600536809030499/dn2477Isup2.hkl

Additional supplementary materials:  crystallographic information; 3D view; checkCIF report

## Figures and Tables

**Table 1:** Hydrogen-bond geometry (Å, °)

*D*—H⋯*A*	*D*—H	H⋯*A*	*D*⋯*A*	*D*—H⋯*A*
C23—H23⋯O3^i^	0.93	2.60	3.476 (2)	158
C18—H18*B*⋯*Cg*3^ii^	0.96	2.91	3.612 (2)	131

Symmetry codes: (i) 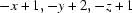; (ii) 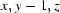.

**Table 2:** Table 2. π-π stacking in the title compound (Å, °)

*CgI*–*CgJ*	*Cg*–*Cg*	α	(*Cg*–*Cg*)_Perp_	(*CgJ*–*CgI*)_Perp_	Slippage	
*Cg*1–*Cg*2^iii^	3.5479 (10)	3.27	3.482	3.483	0.676	

*Cg*1 and *Cg*2 are the centroids of the N1,C1,C6–C9 and C1–C6 rings, respectively, and α is the angle between the corresponding planes. Symmetry code: (iii) 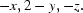

**Fig. 1.**
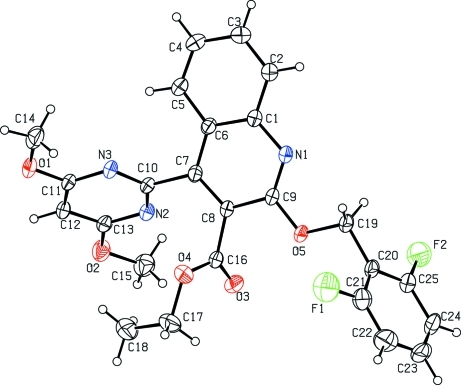
Molecular structure of (I), showing the atom-numbering scheme. Displacement ellipsoids are drawn at the 30% probability level.

**Fig. 2.**
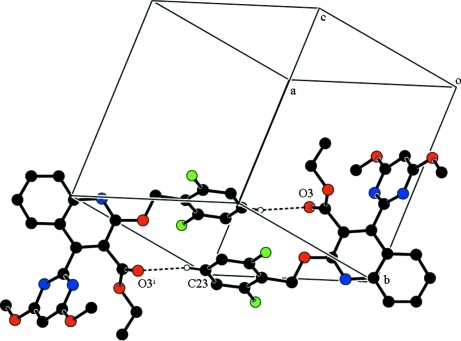
Part of the crystal structure of (I), showing the formation of the pseudo dimer through C-H···O hydrogen bonds. Hydrogen atoms not involved in hydrogen bondings have been omitted for clarity. [Symmetry code: (i) -x+1, -y+2, -z+1]

## supplementary crystallographic information

### Comment

Pyrimidine derivatives have broad biological properties: in particular pyrimidinylbenzoates is a highly effective herbicide with acetohydroxyacid synthase (AHAS) as target (Duggleby & Pang, 2000;). We herein report the crystal structure of one such pyrimidine derivative, the title compound, (I).

In the title compound (Fig.1), the pyrimidine (N2/N3/C10–C13) and difluorobenzyloxy (C20—C25) rings are twisted away from the mid quinoline ring (N1/C1—C9) with the dihedral angles of 54.6 (1)° and 74.1 (1)°, respectively. No other abnormal bond lengths and bond angles were observed in (I) in contrast with its some analogs (Li *et al.*, 2006; Li & Huang, 2007; Li & Wang, 2007).

A weak C—H···O interactions links symmetry related molecules to form a pseudo dimer (Fig.2 and Table 1). Then π-π interactions between the quinoline rings of symmetry related molecules link these dimers into a one-dimensional chain running parallel to the [1 0 1] direction (Table 2). Furthermore, weak C—H···π interactions (Table 1) join these adjacent [101] chains to form a two-dimensional layer running parallel to the (101) plane.

### Experimental

A sulution of ethyl 4-(4,6-dimethoxypyrimidin-2-yl)-2-oxo-1,2-dihydroquinoline -3-carboxylate (0.35 g, 1 mmol) and NaH (60%, dispersion in mineral oil) (0.05 g, 1.2 mmol) in 10 ml *N*,*N*-dimethylformamide(DMF) was stirred at 273k for 0.5 h, and 2-(bromomethyl)-1,3-difluorobenzene (0.23 g, 1.1 mmol) was then added for 10 h.The reaction 200 ml of water, extracted with ethyl acetate (50 ml × 3), dried with anhydrous magnesium sulfate, and filtered off by suction, and the solvent was evaporated to give the crude product, which was purified by chromatography on silica using petroleum ether/acetone (8:1 *v*/*v*) as eluant to obtain the title compound as a white solid (yield 0.19 g, 47%). The product was recrystallized from an ethanol at room temperature to give crystals suitable for single-crystal X-ray diffraction.

### Refinement

All the H atoms were positioned geometrically, with C–H = 0.93, 0.97 and 0.96 Å for aromatic, methylene and methyl groups, respectively, and constrained to ride on their parent atoms, with *U*_iso_(H) = 1.2*U*_eq_(C) or 1.5*U*_eq_(methyl C).

### Crystal data

**Table:**

C_25_H_21_F_2_N_3_O_5_	*Z* = 2
*M**_r_* = 481.45	*F*(000) = 500
Triclinic, *P*1	*D*_x_ = 1.390 Mg m^−^^3^
Hall symbol: -P 1	Mo *K*α radiation, λ = 0.71073 Å
*a* = 9.6623 (6) Å	Cell parameters from 4454 reflections
*b* = 10.7044 (6) Å	θ = 2.5–27.9°
*c* = 11.3856 (7) Å	µ = 0.11 mm^−^^1^
α = 84.466 (1)°	*T* = 297 K
β = 82.251 (2)°	Block, colourless
γ = 81.394 (1)°	0.20 × 0.20 × 0.10 mm
*V* = 1150.21 (12) Å^3^	

### Data collection

**Table:**

Bruker SMART APEX CCD area-detector diffractometer	4954 independent reflections
Radiation source: fine focus sealed Siemens Mo tube	3541 reflections with *I* > 2σ(*I*)
graphite	*R*_int_ = 0.035
0.3° wide ω exposures scans	θ_max_ = 27.0°, θ_min_ = 1.8°
Absorption correction: multi-scan (*SADABS*; Sheldrick, 1997)	*h* = −12→12
*T*_min_ = 0.959, *T*_max_ = 0.989	*k* = −13→13
12431 measured reflections	*l* = −14→14

### Refinement

**Table:**

Refinement on *F*^2^	Primary atom site location: structure-invariant direct methods
Least-squares matrix: full	Secondary atom site location: difference Fourier map
*R*[*F*^2^ > 2σ(*F*^2^)] = 0.052	Hydrogen site location: inferred from neighbouring sites
*wR*(*F*^2^) = 0.170	H-atom parameters constrained
*S* = 1.06	*w* = 1/[σ^2^(*F*_o_^2^) + (0.1077*P*)^2^ + 0.004*P*] where *P* = (*F*_o_^2^ + 2*F*_c_^2^)/3
4954 reflections	(Δ/σ)_max_ < 0.001
319 parameters	Δρ_max_ = 0.30 e Å^−^^3^
0 restraints	Δρ_min_ = −0.23 e Å^−^^3^

### Special details

**Table:**

Geometry. All e.s.d.'s (except the e.s.d. in the dihedral angle between two l.s. planes) are estimated using the full covariance matrix. The cell e.s.d.'s are taken into account individually in the estimation of e.s.d.'s in distances, angles and torsion angles; correlations between e.s.d.'s in cell parameters are only used when they are defined by crystal symmetry. An approximate (isotropic) treatment of cell e.s.d.'s is used for estimating e.s.d.'s involving l.s. planes.
Refinement. Refinement of *F*^2^ against ALL reflections. The weighted *R*-factor *wR* and goodness of fit *S* are based on *F*^2^, conventional *R*-factors *R* are based on *F*, with *F* set to zero for negative *F*^2^. The threshold expression of *F*^2^ > σ(*F*^2^) is used only for calculating *R*-factors(gt) *etc*. and is not relevant to the choice of reflections for refinement. *R*-factors based on *F*^2^ are statistically about twice as large as those based on *F*, and *R*- factors based on ALL data will be even larger.

### Fractional atomic coordinates and isotropic or equivalent isotropic displacement parameters (Å^2^)

**Table:**

	*x*	*y*	*z*	*U*_iso_*/*U*_eq_	
C1	−0.11250 (16)	1.02922 (15)	0.13717 (14)	0.0399 (4)	
C2	−0.20285 (19)	1.12507 (18)	0.08156 (17)	0.0518 (4)	
H2	−0.1963	1.2096	0.0892	0.062*	
C3	−0.3002 (2)	1.09504 (19)	0.01640 (17)	0.0559 (5)	
H3	−0.3596	1.1593	−0.0200	0.067*	
C4	−0.31136 (19)	0.96834 (19)	0.00409 (17)	0.0535 (5)	
H4	−0.3772	0.9490	−0.0414	0.064*	
C5	−0.22682 (18)	0.87332 (17)	0.05793 (15)	0.0467 (4)	
H5	−0.2358	0.7894	0.0496	0.056*	
C6	−0.12486 (16)	0.90099 (15)	0.12685 (13)	0.0382 (4)	
C7	−0.02903 (16)	0.80657 (15)	0.18258 (14)	0.0379 (4)	
C8	0.07269 (16)	0.84312 (14)	0.23904 (14)	0.0378 (4)	
C9	0.07460 (16)	0.97547 (15)	0.24412 (14)	0.0397 (4)	
C10	−0.03328 (16)	0.66870 (15)	0.17606 (13)	0.0378 (4)	
C11	−0.15329 (17)	0.50141 (15)	0.20841 (15)	0.0439 (4)	
C12	−0.03457 (18)	0.42061 (16)	0.17093 (16)	0.0474 (4)	
H12	−0.0349	0.3339	0.1700	0.057*	
C13	0.08520 (18)	0.47704 (15)	0.13469 (15)	0.0428 (4)	
C14	−0.3984 (2)	0.5352 (2)	0.2799 (2)	0.0682 (6)	
H14A	−0.3829	0.5797	0.3452	0.102*	
H14B	−0.4753	0.4874	0.3041	0.102*	
H14C	−0.4207	0.5951	0.2145	0.102*	
C15	0.3280 (2)	0.4621 (2)	0.0603 (2)	0.0650 (5)	
H15A	0.3116	0.5246	−0.0044	0.097*	
H15B	0.4055	0.3993	0.0354	0.097*	
H15C	0.3501	0.5021	0.1261	0.097*	
C16	0.17828 (19)	0.75111 (15)	0.29953 (15)	0.0439 (4)	
C17	0.1993 (3)	0.5997 (2)	0.4653 (2)	0.0774 (7)	
H17A	0.1614	0.6036	0.5484	0.093*	
H17B	0.2941	0.6220	0.4559	0.093*	
C18	0.2047 (3)	0.4705 (2)	0.4309 (2)	0.0838 (7)	
H18A	0.1105	0.4507	0.4343	0.126*	
H18B	0.2555	0.4119	0.4845	0.126*	
H18C	0.2516	0.4643	0.3514	0.126*	
C19	0.17799 (19)	1.13573 (16)	0.31615 (18)	0.0514 (5)	
H19A	0.0887	1.1728	0.3567	0.062*	
H19B	0.1933	1.1807	0.2384	0.062*	
C20	0.29534 (18)	1.14504 (15)	0.38676 (16)	0.0445 (4)	
C21	0.2822 (2)	1.12069 (18)	0.50781 (18)	0.0598 (5)	
C22	0.3845 (3)	1.1322 (2)	0.5770 (2)	0.0815 (8)	
H22	0.3701	1.1150	0.6590	0.098*	
C23	0.5084 (3)	1.1696 (2)	0.5223 (3)	0.0841 (8)	
H23	0.5792	1.1781	0.5676	0.101*	
C24	0.5287 (2)	1.1944 (2)	0.4020 (3)	0.0767 (7)	
H24	0.6129	1.2195	0.3648	0.092*	
C25	0.4223 (2)	1.18172 (18)	0.33639 (19)	0.0555 (5)	
F1	0.16058 (16)	1.08091 (16)	0.55995 (13)	0.0978 (5)	
F2	0.44154 (15)	1.20738 (14)	0.21759 (13)	0.0900 (5)	
N1	−0.01274 (14)	1.06487 (12)	0.19857 (12)	0.0431 (3)	
N2	0.08787 (14)	0.60077 (13)	0.13662 (12)	0.0415 (3)	
N3	−0.15616 (14)	0.62592 (13)	0.21236 (12)	0.0435 (3)	
O1	−0.27320 (13)	0.45076 (12)	0.24386 (13)	0.0572 (4)	
O2	0.20379 (13)	0.40262 (12)	0.09621 (12)	0.0580 (4)	
O3	0.30275 (14)	0.73618 (13)	0.27045 (13)	0.0606 (4)	
O4	0.11257 (15)	0.68999 (12)	0.39364 (12)	0.0596 (4)	
O5	0.17624 (12)	1.00344 (10)	0.30413 (11)	0.0485 (3)	

### Atomic displacement parameters (Å^2^)

**Table:**

	*U*^11^	*U*^22^	*U*^33^	*U*^12^	*U*^13^	*U*^23^
C1	0.0371 (8)	0.0391 (9)	0.0455 (9)	−0.0126 (7)	−0.0069 (7)	0.0001 (7)
C2	0.0490 (10)	0.0417 (9)	0.0676 (12)	−0.0110 (8)	−0.0175 (9)	0.0032 (8)
C3	0.0509 (11)	0.0558 (11)	0.0623 (11)	−0.0051 (9)	−0.0198 (9)	0.0044 (9)
C4	0.0453 (10)	0.0634 (12)	0.0571 (11)	−0.0112 (9)	−0.0203 (8)	−0.0056 (9)
C5	0.0449 (9)	0.0472 (10)	0.0533 (10)	−0.0138 (7)	−0.0150 (7)	−0.0063 (7)
C6	0.0352 (8)	0.0413 (9)	0.0410 (8)	−0.0122 (7)	−0.0073 (6)	−0.0027 (6)
C7	0.0390 (8)	0.0360 (8)	0.0419 (8)	−0.0137 (6)	−0.0069 (6)	−0.0029 (6)
C8	0.0378 (8)	0.0329 (8)	0.0464 (9)	−0.0124 (6)	−0.0108 (7)	−0.0022 (6)
C9	0.0385 (8)	0.0357 (8)	0.0495 (9)	−0.0154 (7)	−0.0119 (7)	−0.0020 (7)
C10	0.0402 (8)	0.0360 (8)	0.0422 (8)	−0.0126 (6)	−0.0144 (6)	−0.0041 (6)
C11	0.0468 (10)	0.0412 (9)	0.0512 (9)	−0.0201 (7)	−0.0213 (7)	0.0019 (7)
C12	0.0550 (10)	0.0336 (8)	0.0616 (11)	−0.0168 (8)	−0.0227 (8)	−0.0049 (7)
C13	0.0476 (9)	0.0376 (9)	0.0492 (9)	−0.0106 (7)	−0.0182 (7)	−0.0085 (7)
C14	0.0474 (11)	0.0685 (14)	0.0941 (16)	−0.0252 (10)	−0.0097 (10)	−0.0055 (11)
C15	0.0515 (11)	0.0654 (13)	0.0776 (14)	−0.0086 (10)	−0.0020 (10)	−0.0108 (11)
C16	0.0513 (10)	0.0331 (8)	0.0542 (10)	−0.0139 (7)	−0.0213 (8)	−0.0048 (7)
C17	0.1054 (18)	0.0602 (13)	0.0727 (14)	−0.0146 (12)	−0.0449 (13)	0.0172 (11)
C18	0.1047 (19)	0.0519 (13)	0.1008 (18)	−0.0081 (12)	−0.0420 (15)	0.0019 (12)
C19	0.0514 (10)	0.0326 (9)	0.0774 (12)	−0.0126 (7)	−0.0247 (9)	−0.0068 (8)
C20	0.0461 (9)	0.0311 (8)	0.0616 (11)	−0.0103 (7)	−0.0155 (8)	−0.0101 (7)
C21	0.0686 (13)	0.0479 (11)	0.0653 (12)	−0.0085 (9)	−0.0097 (10)	−0.0141 (9)
C22	0.120 (2)	0.0638 (14)	0.0692 (14)	−0.0051 (14)	−0.0414 (14)	−0.0200 (11)
C23	0.1000 (19)	0.0523 (13)	0.119 (2)	−0.0128 (13)	−0.0703 (17)	−0.0179 (13)
C24	0.0579 (13)	0.0531 (12)	0.131 (2)	−0.0246 (10)	−0.0378 (13)	−0.0045 (13)
C25	0.0548 (11)	0.0440 (10)	0.0733 (13)	−0.0174 (8)	−0.0191 (9)	−0.0007 (9)
F1	0.0910 (10)	0.1135 (12)	0.0842 (10)	−0.0252 (9)	0.0183 (8)	−0.0065 (8)
F2	0.0820 (9)	0.1069 (12)	0.0810 (9)	−0.0321 (8)	−0.0064 (7)	0.0174 (8)
N1	0.0414 (7)	0.0344 (7)	0.0575 (9)	−0.0139 (6)	−0.0137 (6)	0.0002 (6)
N2	0.0443 (8)	0.0374 (7)	0.0475 (8)	−0.0127 (6)	−0.0122 (6)	−0.0066 (6)
N3	0.0433 (8)	0.0389 (8)	0.0539 (8)	−0.0171 (6)	−0.0131 (6)	−0.0040 (6)
O1	0.0496 (7)	0.0471 (7)	0.0822 (9)	−0.0258 (6)	−0.0164 (6)	−0.0001 (6)
O2	0.0518 (8)	0.0441 (7)	0.0809 (9)	−0.0077 (6)	−0.0085 (6)	−0.0169 (6)
O3	0.0431 (7)	0.0551 (8)	0.0882 (10)	−0.0084 (6)	−0.0251 (6)	−0.0016 (7)
O4	0.0696 (9)	0.0501 (8)	0.0614 (8)	−0.0137 (6)	−0.0221 (7)	0.0121 (6)
O5	0.0503 (7)	0.0322 (6)	0.0715 (8)	−0.0135 (5)	−0.0286 (6)	−0.0050 (5)

### Geometric parameters (Å, °)

**Table:**

C1—N1	1.381 (2)	C14—H14C	0.9600
C1—C2	1.404 (2)	C15—O2	1.435 (2)
C1—C6	1.413 (2)	C15—H15A	0.9600
C2—C3	1.365 (2)	C15—H15B	0.9600
C2—H2	0.9300	C15—H15C	0.9600
C3—C4	1.398 (3)	C16—O3	1.194 (2)
C3—H3	0.9300	C16—O4	1.336 (2)
C4—C5	1.358 (3)	C17—O4	1.446 (2)
C4—H4	0.9300	C17—C18	1.465 (3)
C5—C6	1.419 (2)	C17—H17A	0.9700
C5—H5	0.9300	C17—H17B	0.9700
C6—C7	1.427 (2)	C18—H18A	0.9600
C7—C8	1.366 (2)	C18—H18B	0.9600
C7—C10	1.492 (2)	C18—H18C	0.9600
C8—C9	1.426 (2)	C19—O5	1.4386 (18)
C8—C16	1.498 (2)	C19—C20	1.496 (2)
C9—N1	1.295 (2)	C19—H19A	0.9700
C9—O5	1.3525 (18)	C19—H19B	0.9700
C10—N2	1.329 (2)	C20—C21	1.370 (3)
C10—N3	1.336 (2)	C20—C25	1.378 (3)
C11—N3	1.334 (2)	C21—F1	1.352 (2)
C11—O1	1.3487 (19)	C21—C22	1.370 (3)
C11—C12	1.374 (2)	C22—C23	1.370 (4)
C12—C13	1.381 (2)	C22—H22	0.9300
C12—H12	0.9300	C23—C24	1.363 (4)
C13—N2	1.331 (2)	C23—H23	0.9300
C13—O2	1.341 (2)	C24—C25	1.380 (3)
C14—O1	1.435 (2)	C24—H24	0.9300
C14—H14A	0.9600	C25—F2	1.347 (2)
C14—H14B	0.9600		
			
N1—C1—C2	118.10 (14)	H15A—C15—H15C	109.5
N1—C1—C6	122.54 (14)	H15B—C15—H15C	109.5
C2—C1—C6	119.36 (14)	O3—C16—O4	124.76 (16)
C3—C2—C1	120.49 (17)	O3—C16—C8	125.61 (16)
C3—C2—H2	119.8	O4—C16—C8	109.61 (15)
C1—C2—H2	119.8	O4—C17—C18	111.28 (18)
C2—C3—C4	120.44 (17)	O4—C17—H17A	109.4
C2—C3—H3	119.8	C18—C17—H17A	109.4
C4—C3—H3	119.8	O4—C17—H17B	109.4
C5—C4—C3	120.58 (16)	C18—C17—H17B	109.4
C5—C4—H4	119.7	H17A—C17—H17B	108.0
C3—C4—H4	119.7	C17—C18—H18A	109.5
C4—C5—C6	120.53 (16)	C17—C18—H18B	109.5
C4—C5—H5	119.7	H18A—C18—H18B	109.5
C6—C5—H5	119.7	C17—C18—H18C	109.5
C1—C6—C5	118.59 (14)	H18A—C18—H18C	109.5
C1—C6—C7	117.55 (13)	H18B—C18—H18C	109.5
C5—C6—C7	123.81 (14)	O5—C19—C20	107.44 (13)
C8—C7—C6	119.30 (14)	O5—C19—H19A	110.2
C8—C7—C10	119.15 (14)	C20—C19—H19A	110.2
C6—C7—C10	121.50 (13)	O5—C19—H19B	110.2
C7—C8—C9	118.12 (14)	C20—C19—H19B	110.2
C7—C8—C16	123.18 (14)	H19A—C19—H19B	108.5
C9—C8—C16	118.67 (13)	C21—C20—C25	115.06 (17)
N1—C9—O5	120.62 (13)	C21—C20—C19	121.87 (17)
N1—C9—C8	125.03 (14)	C25—C20—C19	123.05 (17)
O5—C9—C8	114.34 (13)	F1—C21—C20	116.70 (18)
N2—C10—N3	126.87 (14)	F1—C21—C22	119.1 (2)
N2—C10—C7	115.84 (14)	C20—C21—C22	124.1 (2)
N3—C10—C7	117.27 (14)	C23—C22—C21	118.3 (2)
N3—C11—O1	118.56 (15)	C23—C22—H22	120.8
N3—C11—C12	123.87 (15)	C21—C22—H22	120.8
O1—C11—C12	117.57 (15)	C24—C23—C22	120.5 (2)
C11—C12—C13	115.41 (15)	C24—C23—H23	119.8
C11—C12—H12	122.3	C22—C23—H23	119.8
C13—C12—H12	122.3	C23—C24—C25	118.9 (2)
N2—C13—O2	119.06 (15)	C23—C24—H24	120.5
N2—C13—C12	123.08 (16)	C25—C24—H24	120.5
O2—C13—C12	117.86 (15)	F2—C25—C20	117.91 (16)
O1—C14—H14A	109.5	F2—C25—C24	119.04 (19)
O1—C14—H14B	109.5	C20—C25—C24	123.0 (2)
H14A—C14—H14B	109.5	C9—N1—C1	117.38 (13)
O1—C14—H14C	109.5	C10—N2—C13	115.82 (14)
H14A—C14—H14C	109.5	C11—N3—C10	114.94 (14)
H14B—C14—H14C	109.5	C11—O1—C14	117.78 (14)
O2—C15—H15A	109.5	C13—O2—C15	117.34 (14)
O2—C15—H15B	109.5	C16—O4—C17	117.21 (17)
H15A—C15—H15B	109.5	C9—O5—C19	116.23 (12)
O2—C15—H15C	109.5		
			
N1—C1—C2—C3	177.81 (17)	C25—C20—C21—F1	−178.15 (16)
C6—C1—C2—C3	−1.1 (3)	C19—C20—C21—F1	3.6 (3)
C1—C2—C3—C4	0.0 (3)	C25—C20—C21—C22	0.7 (3)
C2—C3—C4—C5	0.8 (3)	C19—C20—C21—C22	−177.59 (18)
C3—C4—C5—C6	−0.5 (3)	F1—C21—C22—C23	178.40 (19)
N1—C1—C6—C5	−177.47 (15)	C20—C21—C22—C23	−0.4 (3)
C2—C1—C6—C5	1.4 (2)	C21—C22—C23—C24	−0.1 (3)
N1—C1—C6—C7	−0.2 (2)	C22—C23—C24—C25	0.2 (4)
C2—C1—C6—C7	178.70 (15)	C21—C20—C25—F2	−179.82 (17)
C4—C5—C6—C1	−0.6 (3)	C19—C20—C25—F2	−1.5 (3)
C4—C5—C6—C7	−177.72 (16)	C21—C20—C25—C24	−0.5 (3)
C1—C6—C7—C8	−2.4 (2)	C19—C20—C25—C24	177.74 (17)
C5—C6—C7—C8	174.76 (15)	C23—C24—C25—F2	179.4 (2)
C1—C6—C7—C10	−179.66 (14)	C23—C24—C25—C20	0.1 (3)
C5—C6—C7—C10	−2.5 (2)	O5—C9—N1—C1	179.15 (14)
C6—C7—C8—C9	2.7 (2)	C8—C9—N1—C1	−2.2 (2)
C10—C7—C8—C9	180.00 (14)	C2—C1—N1—C9	−176.48 (15)
C6—C7—C8—C16	−179.72 (15)	C6—C1—N1—C9	2.4 (2)
C10—C7—C8—C16	−2.4 (2)	N3—C10—N2—C13	−0.7 (2)
C7—C8—C9—N1	−0.4 (2)	C7—C10—N2—C13	177.77 (13)
C16—C8—C9—N1	−178.08 (16)	O2—C13—N2—C10	−179.84 (14)
C7—C8—C9—O5	178.39 (14)	C12—C13—N2—C10	0.0 (2)
C16—C8—C9—O5	0.7 (2)	O1—C11—N3—C10	179.87 (14)
C8—C7—C10—N2	−50.7 (2)	C12—C11—N3—C10	0.3 (2)
C6—C7—C10—N2	126.58 (16)	N2—C10—N3—C11	0.5 (2)
C8—C7—C10—N3	127.95 (16)	C7—C10—N3—C11	−177.91 (13)
C6—C7—C10—N3	−54.8 (2)	N3—C11—O1—C14	2.2 (2)
N3—C11—C12—C13	−0.9 (2)	C12—C11—O1—C14	−178.28 (16)
O1—C11—C12—C13	179.53 (14)	N2—C13—O2—C15	0.7 (2)
C11—C12—C13—N2	0.8 (2)	C12—C13—O2—C15	−179.17 (16)
C11—C12—C13—O2	−179.41 (15)	O3—C16—O4—C17	0.2 (3)
C7—C8—C16—O3	115.1 (2)	C8—C16—O4—C17	−178.10 (15)
C9—C8—C16—O3	−67.3 (2)	C18—C17—O4—C16	−96.3 (2)
C7—C8—C16—O4	−66.5 (2)	N1—C9—O5—C19	1.6 (2)
C9—C8—C16—O4	111.05 (16)	C8—C9—O5—C19	−177.26 (14)
O5—C19—C20—C21	−77.8 (2)	C20—C19—O5—C9	178.85 (14)
O5—C19—C20—C25	104.00 (19)		

### Hydrogen-bond geometry (Å, °)

**Table:**

*D*—H···*A*	*D*—H	H···*A*	*D*···*A*	*D*—H···*A*
C5—H5···N3	0.93	2.54	3.075 (2)	117
C23—H23···O3^i^	0.93	2.60	3.476 (2)	158
C18—H18B···Cg3^ii^	0.96	2.91	3.612 (2)	131

Symmetry codes: (i) −*x*+1, −*y*+2, −*z*+1; (ii) *x*, *y*−1, *z*.

### Table 2 Table 2. π-π stacking in the title compound (Å, °)

**Table:**

Cg(I)–Cg(J)	Cg–Cg	α	(CgI–CgJ)_Perp_	(CgJ–CgI)_Perp_	Slippage	
*Cg*1–*Cg*2^iii^	3.5479 (10)	3.27	3.482	3.483	0.676	

*Cg*1 and *Cg*2 are the centroids of the N1,C1,C6–C9 and C1–C6 rings, respectively, and α is the angle between the corresponding planes. Symmetry code: (iii) -x, 2-y, -z.

## References

[bb1] Bruker (2001). *SMART* and *SAINT* Bruker AXS Inc., Madison, Wisconsin, USA.

[bb2] Duggleby, R. G. & Pang, S. S. (2000). *J. Biochem. Mol. Biol.* **33**, 1–36.

[bb3] Li, Y. & Huang, G. (2007). *Acta Cryst.* E**63**, o4667.

[bb4] Li, Y. X., Luo, Y. P., Xi, Z., Niu, C. W., He, Y. Z. & Yang, G. F. (2006). *J. Agric. Food Chem.* **54**, 9135–9139. 10.1021/jf061976j 17117801

[bb5] Li, Y.-X. & Wang, Y.-Z. (2007). *Acta Cryst.* E**63**, o873–o874.

[bb6] Sheldrick, G. M. (1997). *SADABS*>. University of Göttingen, Germany.

[bb7] Sheldrick, G. M. (2008). *Acta Cryst.* A**64**, 112–122. 10.1107/S0108767307043930 18156677

[bb8] Spek, A. L. (2009). *Acta Cryst.* D**65**, 148–155. 10.1107/S090744490804362X PMC2631630 19171970

